# Petroleum Hydrocarbon Profiles of Water and Sediment of Algoa Bay, Eastern Cape, South Africa

**DOI:** 10.3390/ijerph14101263

**Published:** 2017-10-20

**Authors:** Abiodun O. Adeniji, Omobola O. Okoh, Anthony I. Okoh

**Affiliations:** 1 SAMRC Microbial Water Quality Monitoring Centre, University of Fort Hare, Alice 5700, South Africa; ookoh@ufh.ac.za (O.O.O.); aokoh@ufh.ac.za (A.I.O.); 2 Department of Chemistry, University of Fort Hare, Alice 5700, South Africa; 3 Applied and Environmental Microbiology Research Group, Department of Biochemistry and Microbiology, University of Fort Hare, Alice 5700, South Africa

**Keywords:** petroleum hydrocarbon, physicochemical properties, diagnostic indices, organic carbon, Algoa Bay, gas chromatography–flame ionization detector

## Abstract

Petroleum hydrocarbon profiles of water and sediment samples of Algoa Bay in the Eastern Cape Province of South Africa were assessed using standard analytical procedures. Water (from surface and bottom levels) and sediment samples were collected from five locations in the bay from February to June 2016. Extraction of the petroleum hydrocarbons from the water and sediment samples collected was achieved using liquid-liquid and Soxhlet extraction techniques, respectively, followed by column clean up. Target compounds were analytically determined with gas chromatography–flame ionization detector (GC-FID) and quantified by integrating the areas of both the resolved and unresolved components. Physicochemical properties of the water samples were also determined on site using a SeaBird 19plusV2 CTD SBE 55 device. Estimated limit of detection, limit of quantitation and relative standard deviation for the 35 *n*-alkane standards ranged from 0.06 to 0.13 μg/L, 0.30 to 0.69 μg/L and 3.61 to 8.32%, respectively. Results showed that total petroleum hydrocarbon (TPH) varied from 45.07 to 307 μg/L in the water and 0.72 to 27.03 mg/kg in the sediments. The mean concentrations of TPH in both the water and sediment samples from Algoa Bay revealed a slight level of pollution. The diagnostic indices used showed that the hydrocarbons in the area were from both biogenic and anthropogenic sources. Hence, there is need for adequate regulation and control of all activities contributing to the levels of petroleum hydrocarbon in the marine environment for the safety of human, aquatic and wild lives in the area.

## 1. Introduction

Large quantities of sewage, dredged spoils, stormwater, spilled oil, municipal and industrial wastes are discharged into the coastal water bodies through their estuaries with little or no measure of treatment, especially in the developing countries [1,2]. Such discharges are mainly from the coastal urban areas and although at low concentrations may not pose any immediate threat, but could trigger a great challenge over a long period of time [3].

Petroleum hydrocarbons (PHCs) are one of the notable organic contaminants found in the organic wastes [4,5]. They have been largely utilized as tools for detecting the sources of petroleum residues in the marine environments [6,7,8]. Oil spills from land-based sources (refineries, storage facilities, municipal and industrial wastes, river runoff etc.) and transportation activities (tanker oil transportation and shipping) are reportedly more damaging in cold regions than in the warmer climates [9] and its impacts on aquaculture could be very severe because oil has a tendency to bio-accumulate in the tissues of fish, molluscs, mussels and other mammals [10]. 

PHCs are usually carried into the sea in the form of solutions, either as stormwaters, urban runoffs, domestic wastes or industrial effluents, but only a small portion of the load eventually remains in solution. Instead, they are scavenged from the water column to the bottom sediments through flocculation, sedimentation and coagulation, giving rise to concentrations in the sediment many orders of magnitude higher than in the water column [11,12]. Sediment therefore remains the potential sink for petroleum hydrocarbons and other organic pollutants, and its contamination could represent a very great health hazard for many aquatic organisms that reside in such an ecosystems [13,14]. The most important components of the petroleum hydrocarbons in the aquatic environments are the normal alkanes, combusted hydrocarbons and degraded crude oils [15]. 

Various physical and chemical properties that influence the survival of aquatic organisms in water and sediment include but not limited to the following; temperature, pH, conductivity, dissolved oxygen, salinity, turbidity, chlorophyll, total suspended solids, total dissolved solids, sediment moisture, organic carbon and matter [16,17]. These qualities are highly instrumental to the assessment of the level of damage done to the waterways and their deviation from natural levels can result in ecosystem deterioration [18]. Inflow of municipal effluents, stormwaters and industrial discharges into the rivers, lakes, estuary, bay and oceans as a result of global increase in urbanization and industrialization are channels for serious environmental pollution with relatively high consequences on human health, aquatic ecosystem balance, as well as social and economic development [19,20].

They are the appropriate indicators for the suitability of the water for its various applications and are capable of affecting the biological characteristics of the environmental media [6,21,22,23,24]. The physicochemical characteristics of each organic pollutant in water and sediment will depend on the level and sources of contamination in the environment [25,26]. Therefore, monitoring them in water resources is highly paramount for the protection of human and aquatic lives [23,27]. 

Even though South Africa remains one the largest economies in the continent, not many studies have documented the concentrations of TPH in its environmental resources. A study conducted on the levels of TPH in mussels around the Cape Peninsula in South Africa by fluorescence spectroscopy gave concentrations in the range of 10–100 mg/kg dry weight, which were majorly from harbours, although industrial effluents and sewage were recognized as other possible sources of TPH in the area [28]. In another study by Okonkwo et al. [29], TPH and trace metals in street dusts from the Tshwane Metropolitan Area, South Africa were evaluated. The petroleum hydrocarbons levels which were determined gravimetrically varied from 562 to 2340 mg/kg and 404 to 852 mg/kg in the dust samples collected from petrol service stations and heavy traffic roads, in that order. Recently, the status of TPH in the surface water and sediment of the Buffalo River Estuary in East London, South Africa was evaluated using gas chromatography with flame ionization detection. The findings revealed TPH levels in the range of 8–477 μg/L in the water matrix, and 13–1100 mg/kg in the sediments, indicating a great influence of industrialization and urbanization on the area [30].

Algoa Bay is one of the most important bays in Africa, located in the Eastern Cape Province of South Africa. It is a marine biodiversity hotspot and also a tourism and recreational terminus that contributes immensely to the socio-economic development of the Nelson Mandela Bay Metropolis [31]. The mouth is about 70 km wide [32] and the bay accommodates two large industrial ports (Port of Port Elizabeth and Port of Ngqura), two groups of islands (Cross Island in the southwest and the Bird Island in the northeast), the largest number of the endangered African penguins and supports a small fishing industry. It is an occasional nursery area for some marine invertebrates, southern right whales, great white sharks and other important fishes. No less than eight of the 15 seabird species that reside in South Africa have been identified to be breeding either on its islands or on the nearby shore [31,33,34].

Of the four rivers that flow into the bay, the two most significant in terms of anthropogenic contributions are the Sundays and Swartkops rivers, while the inputs from the Coega and Papenkuil rivers are relatively small [35,36]. From a pollution perspective, Algoa Bay is considered the ultimate sink for the industrial and domestic effluents as well as possible agricultural pollutants coming from the whole Port Elizabeth-Uitenhage-Despatch through Sundays River and to a lesser extent the Swartkops River. The pollutant contribution, especially oil from ship traffic, places additional environmental pressure on this area [33,36], although, major marine pollution threats are restricted to the area between the Cape Recife sewage outfall and the Fishwater Flats sewage outfall, as well as Port Elizabeth Harbour and the Papenkuils River [31,37]. 

Seabirds, and specifically penguins, are vulnerable to oil pollution. Oiled birds are mostly observed on the islands in Algoa Bay and in other marine areas in the country. They were severely affected by the two major oil spills that occurred in the recent past. The 1994 ‘Apollo Sea’ oil spill in which about 2400 tons of oil was released, affected no less than 10,000 African Penguins. Another major experience was that of the ‘MV Treasure’ oil spill 2000, in which about 20,000 African Penguins were contaminated by the release of about 1300 tons of oil [33]. 

Previous pollution studies in the study sites included the assessment of heavy metals, bacteriological features, organochlorine and polychlorinated biphenyls levels in the Swartkops estuary [37]. Snapshot survey of the surface water was carried out by Klages and Bornman [32,33] to determine the status of oil and grease, total petroleum hydrocarbons (TPHs) and polycyclic aromatic hydrocarbons (PAHs) in the water column. The research findings revealed that oil and grease, as well as PAHs levels were higher in the vicinity of St. Croix Island [38], albeit it was variable over time [32] but no such measurements were conducted in the sediment till date [33]. 

The aims of this study are therefore to investigate the pollution status of Algoa Bay by determining the physicochemical properties of the water, concentrations of the aliphatic and total petroleum hydrocarbons in both the water and sediment of Algoa Bay and also to identify the possible sources of contaminants using various ratios and indexes on the n-alkanes. 

## 2. Materials and Methods

### 2.1. Study Area

**Figure 1:**
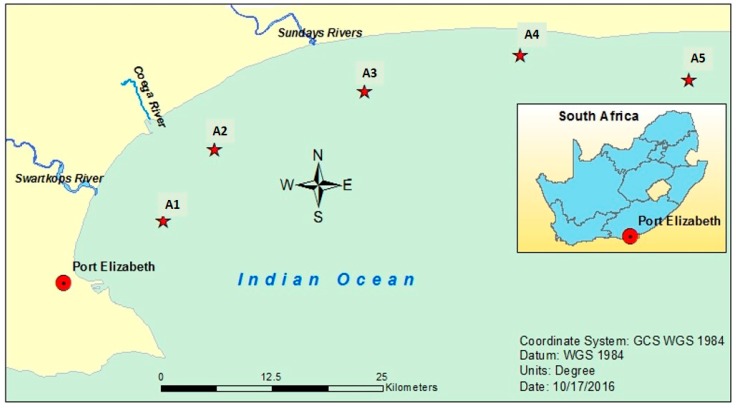
Map of Algoa Bay.

Algoa Bay (latitude: 33°47′34.79″ S and longitude: 25°46′6.59″ E) is located adjacent the large industrial city of Port Elizabeth in the Eastern Cape Province, South Africa, about 425 miles East of the Cape of Good Hope, at the meeting point of two crucial oceanic systems (the Agulhas and the Benguela currents) [39]. The depth in all the sampling locations was fairly constant, with an average of 30 m. The bay receives many domestic, urban, agricultural and industrial discharges through its influent rivers [33]. Five sampling stations were selected and coded as A1, A2, A3, A4 and A5 for convenience as shown in Table 1 below. Figure 1 also shows the map of Algoa Bay, Eastern Cape, South Africa.

### 2.2. Chemicals and Sample Collection

Standard mixtures of *n*-alkanes (C_8_–C_40_; 500 μg/mL each) and 1-chlorooctadecane (250 mg) were sourced from AccuStandard (New Haven, CT, USA). Analytical grade reagents (>98% purity), high performance liquid chromatography (HPLC) grade solvents, anhydrous sodium sulphate and silica gel (70/230 Mesh ASTM) used for this work were purchased from Merck KGaA (Darmstadt, Germany). Glassware was soaked in nitric acid (10%) before washing with liquid soap, then rinsed subsequently with double distilled water and acetone in succession, drained and dried overnight in an air-circulated oven at 105 °C. All the sampling bottles and vials used in this research work were covered with PTFE lined lids. 

Duplicate samples were collected from five stations on the bay. The sampling period was between February and June, 2016 covering three seasons (summer, autumn and winter), although no sample was collected in April on account of unfavourable weather conditions. Water samples (500 mL each) were collected both from the surface (100 mm below) and bottom levels (approximately 25 m depth) using a SeaBird 19plusV2 CTD SBE 55 Carousel (Sea-Bird Scientific, Bellevue, WA, USA) equipped with six 4L Niskin bottles into the pre-cleaned amber bottles whereas sediment samples (1 kg each) were collected from the bay using stainless steel cone dredge into wide-mouth bottles. Acidification of the water samples to pH < 2 was achieved using 6 M hydrochloric acid and they were immediately transported on ice at temperature below 4 °C to the laboratory for chemical analysis [45,46].

### 2.3. Physicochemical Analyses of the Samples

A SeaBird 19plusV2 CTD multi-parameter probe was used for the in situ measurement of pH, temperature, electrical conductivity, dissolved oxygen, salinity and chlorophyll of the water samples on site. Physicochemical properties of the sediment samples including moisture, organic carbon (OC) and organic matter (OM) contents were determined by a gravimetric method [47].

### 2.4. Extraction of Petroleum Hydrocarbon from Water and Sediment Samples

Water samples (500 mL) were extracted three times using separatory funnel with 20 mL portions of *n*-hexane after 1 mL of 10 μg/mL surrogate standard (1-chlorooctadecane) was spiked into each sample. The extracts were combined, dried with anhydrous sodium sulphate and concentrated using a rotary evaporator at 35 °C under reduced pressure to about 2 mL [48,49]. Sediment samples were air-dried for about 5 days and powdered before extraction. Sufficient quantity of anhydrous sodium sulphate (Na_2_SO_4_) was mixed with 10 g of the dried sample for further removal of moisture, spiked with 1 mL of 10 μg/mL surrogate standard and extracted with 200 mL dichloromethane in a Soxhlet extractor for 24 h. The extract was run through a glass funnel containing anhydrous sodium sulphate, concentrated in a rotary evaporator and solvent exchanged to *n*-hexane, ready for cleanup [50]. 

### 2.5. Silica Gel Cleanup and Separation

Both the water and sediment extracts were cleaned up in a chromatographic column (10 mm i.d. × 30 cm) packed with the slurry prepared from 10 g activated silica gel and 2 cm anhydrous Na_2_SO_4_ layer on top. The sample was eluted using 20 mL of *n*-pentane, concentrated and solvent exchanged to *n*-hexane. A blank sample was processed the same way for quality assurance [51].

### 2.6. Gas Chromatography Analysis and Quantitation

The concentrates were analytically determined by gas chromatography (Agilent 7820A GC, Agilent, Santa Clara, CA, USA) coupled with flame ionization detection, using a HP-5 fused silica capillary column (30 m × 0.32 mm i.d. × 0.25 μm film thickness), injecting 1 μL sample in splitless mode at 300 °C. The carrier gas was helium at flow rate of 1.75 mL/min, average velocity of 29.47 cm/s and the detector temperature was 300 °C. The column temperature was set at 40 °C for 1 min and then increased to 320 °C at 7 °C/min [52,53,54]. 

Working standards solutions for both the alkanes and the surrogate (1-chlorooctadecane) were prepared from the stock solutions and kept in amber bottles at <4 °C. The calibration standards in the range of 0.05–20 μg/mL were prepared with *n*-hexane and were used for the calibration of the instrument. The Agilent Chemstation chromatography software was used to generate average response factor for each analyte from the calibration curves plotted which were linear with correlation coefficients ranging from 0.9846 to 0.9919. The approximate linearity obtained for all the analytes were within the acceptable range of R^2^ ≥ 0.990 [25]. As shown in the Table 2, the response factor of nC_15_ was used for the quantification of the unresolved peaks according to the method of Luan and Szelewski [55]. Integration of TPH using the software was achieved with baseline-holding and peak-sum slicing. TPH was thereafter estimated as the total concentration of the n-alkanes eluting from nC9 to nC36 with addition of the UCM [25,55]. 

### 2.7. Quality Control

All reagents and solvents used were analytical and HPLC grades, correspondingly. Samples were routinely analyzed in duplicates with blanks and spiked samples but no interference was found in all the blanks determined. Limit of detection (LOD) for *n*-alkanes was estimated using eight replicate injections of a middle level calibration standard [56,57]. The LOD was calculated by multiplying the “t” value at 99% confidence level with the instrument response and the values obtained were in the range of 0.06–0.13 μg/L. The precision of the instrument which was estimated as the relative standard deviation (RSD) were generally less than the maximum limit of 25% [58,59], varying from 3.61 to 8.32% for the *n*-alkanes. The efficiency of the method was assessed from the recoveries of the spiked samples at concentration level of 20 μg/L and results were largely between 76% and 137%, with an average of 88% and 87% for the water and sediment samples, respectively. Likewise, the recovery of 1-chlorooctadecane added to all the water and sediment samples were between 44% and 96%, which were within the acceptable range of 40–140% for hydrocarbons [58].

### 2.8. Data Analysis

Statistical analysis of all the results was carried out with IBM SPSS version 20 (IBM, Armonk, NY, USA). One way ANOVA was computed for multiple groups and standard errors for individual group of data. Relationship between groups was compared with correlation and the significance was defined as *p* < 0.05 [8]. 

## 3. Results and Discussion

### 3.1. Physicochemical Properties of the Surface and Bottom Water of Algoa Bay

The physicochemical properties of water samples collected from the surface and bottom levels of the bay are summarized in Table 3. Hydrogen ion concentration (pH) is an important water quality parameter that influences both the biological and chemical processes within the water body [60]. It is temperature dependent and can be used to assess the level of effluent discharge into an environment. The pH of most natural waters are in the range of 6.0–8.5, higher values could be obtained from eutrophic and salty water while lower values are usually from dilute water containing high level of organic materials [61,62]. Water temperature is also an important quality parameter, which is largely influenced by many factors including time of the day, season, latitude, air circulation, depth and flow of the water body [60]. Increase in temperature increases rate of chemical reactions in the water body, alongside volatilization and evaporation of substances, but decreases the solubility of gases (including oxygen) in the aquatic systems [61]. 

The pH obtained in this study ranged from 8.3 to 8.9 (mean = 8.6 ± 0.02) and 8.2 to 8.8 (mean = 8.5 ± 0.001) at the surface and bottom levels correspondingly. Generally, pH increases from summer to winter. The pH at the surface level were higher than at the bottom, although they were all within the Australian and New Zealand permissible range of 5.0–9.0 for Fresh and Marine Water Quality, indicating that the water body is likely to be healthy [63]. No significant spatial difference was observed across the sampling stations, neither was there any positive correlation established between pH and TPH in the two matrices (Table 4).

Temperature in this study varied from 17 to 22 °C (mean = 19 ± 0.01) in the surface water and 15 to 19 °C (mean = 17 ± 0.01) in the bottom water and all were within the South African guideline range of 15–35 °C for coastal and recreational waters [64]. The sea surface temperature range was in agreement with previous report [39]. Unlike pH, there was a general decrease from summer to winter at the surface but a noticeable fluctuation at the bottom was observed. The temperature at the surface level were generally higher than at the bottom, explaining the negative correlation with depth (r = −0.648, *p* < 0.01) as shown in Table 4. Warm water usually floats, owing to its lower density while the cold one sinks, especially when salinity is higher (http://oceanexplorer.noaa.gov/facts/temp-vary.html). The swell around the country emanates from storms in the Southern Ocean, as well as the tough, native westerly winds [39]. The temperatures obtained across the sampling locations did not reveal any significant difference. 

Conductivity is the ability of a solution to conduct electrical current. It is related to the concentrations of the major ions, temperature and total dissolved solids in the water body [65]. It roughly indicates the mineral content of the water and could be extremely higher in polluted water or any that receives large quantity of urban run-off [60]. Comparatively, conductivity is usually higher in salty water than freshwater [61]. The conductivity of Algoa bay water ranged from 45.39 to 49.9 μS/m in the surface water and 43.07 to 47.73 μS/m in the bottom water (Table 3). At the superficial level, highest conductivity was observed in summer and the lowest in winter, showing a decrease with seasons. It decreases as temperature also decreases [62] and as depth increases. It was generally higher in the surface water than at the bottom and did not reflect any significant spatial variation. However, the bottom water did not show any observable trend.

Conductivity reveals a positive correlation with dissolved oxygen (r = 0.558, *p* < 0.01) but a negative correlation with turbidity (r = −0.341, *p* < 0.05) and depth (r = −0.651, *p* < 0.01) as shown in Table 4. Although, there is no specific guideline available for conductivity, a value as high as 55 μS/m is possible in the seawater depending on the salinity and temperature of the water body [66].

Both turbidity and salinity showed marginal increase in value across the seasons. Highest values were recorded in summer and lowest in winter, especially in the bottom water. Turbidity varied from 1.09 to 1.99 NTU at the surface and 1.82 to 3.93 NTU at the bottom, indicating a significant increase with depth (r = 0.409; *p* < 0.01) (Table 4). The results were generally within the permissible range of 0.5–10 NTU for the estuary and coastal water, published by the Department of Environment and Conservation, New South Wales [67,68]. A significant spatial variation was observed in the turbidity between Alexandria Dune Fields and Woody Cape (F = 2.925, r = 0.031). This may be due to aggregation of marine rubbles along the coast from Port Elizabeth and Coega harbour and re-suspension of sand from the dune fields, causing a rise in the turbidity of water in the area [36,44,69].

Salinity of a water body is a measure of the amount of dissolved salts in it. It is a very important parameter that determines the survival of aquatic plants and animals [61]. Higher salinity may affect the suitability of the water for shellfish and other marine animals. EU directive [79/923/EEC] has specified a range of 12–38 PSU as tolerable limit for salinity in coastal water [70]. Surface water samples collected for this work recorded salinity in the range of 35.13–35.60 PSU throughout the period of the project and 35.16–35.39 PSU at the bottom level. All the values obtained were within the EU permissible range across the sampling stations with no spatial variation observed. The results compared favorably with earlier findings documented from the bay and the variability of the figures was very limited [71,72].

Dissolved oxygen (DO) is another important parameter that influences the survival of the aquatic lives. The presence of organic matter in the aquiver could drastically deplete the dissolved oxygen in the system [60]. DO is inversely related to temperature, as it recorded its lowest in summer and the highest in the winter at the 25 m depth (bottom) but was fluctuating at the surface with no specific trend [70]. The values generally ranged from 5.51 to 8.96 mg/L and 4.43 to 7.76 mg/L at the surface and bottom levels respectively (Table 3), corroborating the negative correlation it has with depth (r = −0.494, *p* < 0.01). Dissolved oxygen are usually lesser in saltwater than freshwater (~20% lesser) because it has a lower 100% air saturation and thus, the saltwater animals have higher tolerance for lower DO concentrations. DO concentration lower than 5 mg/L is very harmful to the aquatic organisms in the system and needed to be controlled [73]. Coastal fish will avoid an area with DO < 3.5 mg/L and invertebrates <2 mg/L, suggesting a lower survival rate generally at lower DO levels [74]. The dissolved oxygen levels in the present study were relatively lower in the bottom water than at the surface. This could be related to the depth, which does not allow for water equilibration with atmospheric oxygen [75]. The mean concentrations at the two water depths were higher than 5 mg/L, thereby suggesting higher degree of subsistence for the marine animals in the aquatic resources.

Chlorophyll is a measure of alga biomass in the aquatic systems. It is available in three forms (*a*, *b* and *c*) and it reveals the trophic status of a water body. The most commonly determined is chlorophyll *a* and its measurement could be affected by the presence of its degradation products (e.g., phaeophytin) in the ecosystems [60]. There is usually a direct relationship between chlorophyll and some environmental factors including temperature, light and the presence of nutrients within the environment [76]. The concentrations obtained in this study were higher at the bottom level (0.64–4.29 μg/L) than at the surface (0.55–2.54 μg/L) (Table 3), affirming the positive correlation between chlorophyll and depth (r = 0.383, *p* < 0.01). The chlorophyll level in Algoa Bay water was <8 μg/L, implying a lower level of nutrients (termed oligotrophic), which is an indication of a low level of pollution in the coastal environment [70].

### 3.2. Total Petroleum Hydrocarbon Levels in the Waters

The seasonal and total mean concentrations of TPH obtained for water samples in this study are summarized in Table 5. The results generally ranged from 45.07 to 273 μg/L in the surface water and 55.72 to 307 μg/L in the bottom water. No particular trend was observed in the concentrations of TPH across the three seasons of study, although values were higher in summer at the two water levels than other seasons. This was consistent with previous findings reported at the study site, in which the organic contaminant levels were extremely low in winter [33]. The highest concentrations recorded in the surface and bottom waters were obtained at Woody Cape in summer (February) and St Croix in autumn (May), respectively. This could be attributed to urban discharges and vehicle emission from Port Elizabeth metropolis, recurrent industrial pollution from the two harbours, Coega Industrial Development Zone (IDZ) and factories in the neighbourhood of Swartkops River [31,33,35,77,78]. This is because the unresolved complex mixture (UCM) which is an indication of contamination from petroleum origin was below the detection limit at the two instances suggesting pollution from other anthropogenic sources [15]. 

However, the occurrence of petroleum hydrocarbon was conspicuously noticeable in June at both Alexandria Dune Fields (surface) and Woody Cape (bottom) with TPH levels of 173.33 μg/L and 69.65 μg/L, including UCM of 59.90 μg/L and 10.35 μg/L respectively. Oil spill in this region is more damaging to the population of marine life and certain seabirds, especially those in the sensitive and endangered category e.g., African penguins and Roseat terns [33,39,79]. The oil could possibly have emanated from increased port, shipping and boating activities in the area [33,80]. Although, South Africa is yet to set any standard limit for TPH in its waters [33], the mean concentrations in this study for the surface (121.52 ± 14 μg/L) and bottom waters (119.79 ± 13.57 μg/L) as shown in Table 5 were found higher than the target value (7 μg/L) for total petroleum hydrocarbon in the Australian and New Zealand Guidelines (Volume 2) for Fresh and Marine Water Quality [63], indicating that the water columns were polluted. 

**Figure 2:**
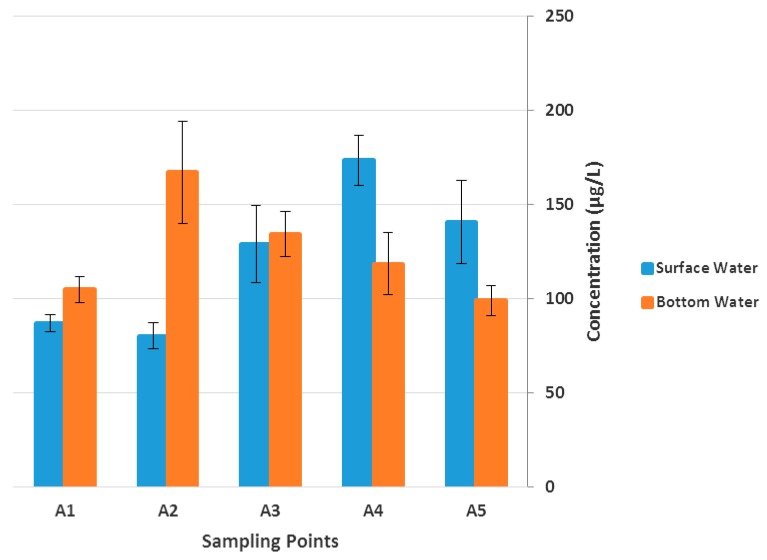
Spatial variation of TPH in the Algoa Bay water samples.

The spatial distribution of TPH in the water columns across the five sampling points as shown in Figure 2 revealed that highest mean concentration in the surface water was detected at Alexandria Dune Fields (173.51 ± 13.38 μg/L), whereas St Croix had the highest at the bottom level (167.14 ± 27.32 μg/L). Generally, the trend at both levels in decreasing order could be given as A4 > A5 > A3 > A1 > A2 (surface) and A2 > A3 > A4 > A1 > A5 (bottom). It is obvious that the TPH level at Sheltered Bay (A1) was generally lower at the two water depths. However, the observed concentrations at Alexandria Dune Field (A4) and St Croix (A2) could be attributed to the contributions from major estuaries that are discharging into the bay (Swartkops, Sundays and Coega), as well as the shipping activities in the area [31,33,39]. 

TPH concentrations in the surface water of Algoa Bay were within the range of values reported for samples in Bohai Bay of China [81], Romanian Black Sea Sector [82] and Kara Sea [83]. Nevertheless, the results were found higher than the levels documented for samples in other regions such as Dungun River basin water, Malaysia [84] and Gulf of Thailand and East Coast of Peninsular Malaysia [85]. 

### 3.3. Total Petroleum Hydrocarbon Levels in the Sediment Samples

TPH concentrations in the sediment samples expressed on dry weight basis ranged from 0.72 to 27.03 mg/kg (Table 6). A steady rise in TPH levels from summer to autumn was observed in the sediment as reported by Maktoof et al. [86] and Karem et al. [87]. This might be due to possible volatilization of hydrocarbons from the sediment, as well as biodegradation of the TPH due to increasing temperature in summer than other seasons. Higher temperature is also known to favour the rate of photochemical decomposition of hydrocarbons in the atmosphere which as a result could bring about the reduced level of the contaminants observed in the season [88]. The microbial breakdown of TPH is usually lower in winter while the rate of emissions (e.g., vehicular, biomass and coal) increases, consequently contributing to higher concentrations of hydrocarbons in that season [89]. 

**Figure 3:**
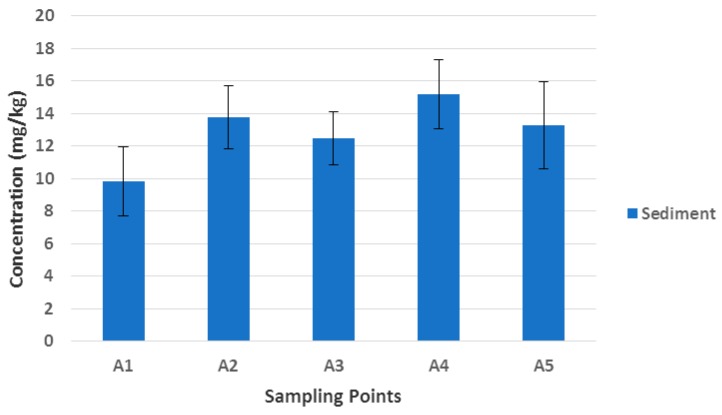
Spatial variation of TPH in the Algoa Bay sediment samples.

The spatial distribution of TPH in this compartment followed a similar trend to that reported above for water matrix. Highest mean concentration was observed at Alexandria Dune Fields (15.18 mg/kg) and the lowest at Sheltered Bay (9.82 mg/kg) as shown in Figure 3. The order of increase in the TPH concentrations is given as follows: A4 > A2 > A5 > A3 > A1. The total mean value (12.77 ± 1.74 mg/kg) recorded was lower than the Nigerian guideline limit of 50 mg/kg for mineral oil in sediment [90,91]. Although, Massoud et al. [92] suggested that sediment TPH in the range of 10–15 mg/kg should be considered as unpolluted and 15–50 mg/kg as slightly polluted. The sediment samples collected from Algoa Bay are therefore by implication between unpolluted and slightly polluted ranges. The elevated results could be a resultant effect of major industrial and shipping activities in the Coega IDZ, including the harbour operational influence at the river mouth, which are capable of increasing the storm water runoff that enters the bay from urban area. Other possible sources include occasional oil spills, vehicular deposits and engine leaks on the asphalt-tarred roads, which are toxic to benthic fauna upon accumulation in the surficial sediment and can bring about developmental malformations, loss of reproductive capacity and several diseases in marine mammals [31]. 

Generally, the levels of sediment TPH in the studied area were found significantly lower than those obtainable in many other coastal environments like Ceuta harbour in North Africa [93]; Musa Bay [94] and Barnegat-Bay-Little Egg Harbor Estuary, USA [95]. However, water from few other locations around the world revealed petroleum hydrocarbons concentrations that were similar to those reported in this study. Examples include those from Southeast Coast of India [2], Todos os Santos Bay, Brazil [96], as well as the coastline and mangroves of the Northern Persian Gulf [97].

### 3.4. Organic Carbon Content of the Sediment

The results of the moisture, organic carbon and organic contents analyses are presented in Table 7. While the moisture contents of the sediments ranged from 13 to 27.96%, the organic carbon and organic matter varied from 1.05 to 2.05% and 1.82 to 3.53% respectively (Table 7). The sediments were largely composed of sand (~95% determined by sieving). Sediments of smaller particles (e.g., muddy sediment) are usually found richer in organic carbon content and they accumulate more organic micro-pollutants than the larger particles [92]. Lower values reported in the present investigation for both the organic carbon content and organic matter of the sediment samples correspond to those previously reported [53]. Statistical analyses revealed a positive correlation between moisture and both the organic carbon and organic matter (*p* < 0.05), however, no correlation existed between TPH and organic carbon (Table 8), implying a very little sorption of petroleum hydrocarbons into the sediment.

### 3.5. Concentrations of n-Alkanes and Source Identification

Concentration of total *n*-alkanes in the surface water column of Algoa bay ranged from 45.07 to 273 μg/L, whereas values in the bottom water ranged from 55.72 to 307 μg/L (Table 5). Similarly, the values obtained in the sediment varied from 0.72 to 27.03 mg/kg (Table 6). The mean sediment value (12.72 ± 1.74 mg/kg) obtained was slightly higher than the permissible level of 5–10 mg/kg dw for coastal sediments [98]. Generally, the higher molecular *n*-alkanes were more abundant than the lighter ones. Likewise, the even numbered aliphatic hydrocarbons (C_18_–C_22_) which is an indication of anthropogenic input were higher in concentration than their odd numbered (C_15_–C_19_) counterparts (from natural sources) (Table 5 and Table 6). The presence of lower molecular *n*-alkanes signals the freshness of hydrocarbon in the environment while the higher molecular ones suggest aged petroleum hydrocarbon of combustion origin [50,54,99]. Therefore, the dominance of some specific even numbered hydrocarbons (C_16_, C_18_, C_20_) over the odd ones (nC_15_, nC_17_, nC_19_) in the study site suggests anthropogenic source pollution. Many ratios and indexes, which relate to the hydrocarbon concentration or even UCM, are employed to differentiate between biogenic and anthropogenic sources of hydrocarbons in the area as shown in the Table 9 [53].

#### 3.5.1. Carbon Preference Index (CPI)

It is a measure of the ratio of the odd to the even numbered carbon hydrocarbons and calculated in different ways. It is used to assess the dominance of natural hydrocarbons over the anthropogenic ones [1,52]. CPI values higher than 1 (mostly in the range of 3–10) depict the presence of biogenic materials (e.g., epicuticular waxes in terrestrial vascular plants or algae) with greater predominance of odd numbered *n*-alkanes (Table 9). Predominance of odd numbered hydrocarbons in the range of nC_15_–nC_21_ indicates the presence of *n*-alkanes from algae or microbial sources while those in the range of nC_23_–nC_31_ are mostly from vascular plants. CPI close to 1 however suggests the presence of hydrocarbons from petrochemical origin [53,100]. The findings of this study gave CPI range of 0–2.15 (Table 6), suggesting the presence of aliphatic hydrocarbons majorly from natural sources. Mathematically, CPI in sediment is expressed as:

CPI_25–33_ = 0.5 × [(C_25_–C_33_)/C_24_–C_32_)] + [C_25_–C_33_)/C_26_–C_34_)] (1)

#### 3.5.2. UCM and Weathering Index (WI)

UCM is the combination of structurally complex isomers and homologous of branched and cyclic hydrocarbons which are difficult to resolve by the capillary columns of gas chromatograph, forming a hump below the resolved compounds [50,101]. Its presence majorly indicates petrogenic contamination [102]. The UCM range in the sediment samples as shown in Table 6 were generally between 0.17 and 0.27 mg/kg, which constitute approximately 2% of the total hydrocarbon concentration in the sediment, suggesting a very low level of petroleum hydrocarbon pollution in the area. Weathering index, which is the ratio of the unresolved components of hydrocarbons to the resolved (U/R) was also calculated for all the samples [103,104]. Generally, U/R > 4 is a pointer to significant presence of crude oil residues (degraded petroleum hydrocarbons) in the sediment [100], which may stay bound for a very long time, resulting in a short term damage of the aquatic lives [6]. The U/R value in this study were generally less than 1, indicating a low level amount of petroleum hydrocarbon contaminants and a healthy environment for the aquatic animals to thrive.

#### 3.5.3. Low Molecular Weight *n*-Alkanes/High Molecular Weights Alkanes (L/H)

The ratio of the low molecular weight (nC_15_ to nC_20_) to the high molecular weights (nC_21_ to nC_34_) *n*-alkanes is also an essential tool for assessing the sources of hydrocarbons in the aquatic environments [54,105]. The ratio ΣLMW/ΣHMW below 1 suggest the predominance of *n*-alkanes from higher plants, aquatic bacteria and marine animals, while values close to 1 indicate *n*-alkanes from petroleum and plankton origin (Table 9). Incidence of fresh oil in the surface water is inferred when the ratio is greater than 2 [8]. The ratios calculated were between 0 and 2.92 with an average of 0.77 ± 0.15 in the sediments, and varied generally from 0.06 to 14.09 in the water column. The findings imply the occurrence of fresh oil pollution in the water compartment. This might be a product of occasional spillage due to shipping or industrial effluent discharge through its tributaries [35].

#### 3.5.4. C_31_/C_19_

Abundance of nC_31_ in the surface water indicates terrestrial biogenic origin, as predominance of nC_19_ implies the presence of *n*-alkanes from marine biogenic sources. The ratio nC_31_/nC_19_ therefore distinguishes hydrocarbons of terrestrial origin from those of marine sources [105]. Values greater than 0.4 indicate the presence of hydrocarbons from non-marine sources [54]. The nC_19_ was not found in the surface water throughout the period and therefore not computed. Notwithstanding, the average concentration of nC_31_ (4.92 ± 1.09 μg/L) suggests a dominance of *n*-alkanes from non-marine sources. Furthermore, the nC_31_/nC_19_ ratio in the sediment samples ranged from 0 to 7.41 with an average of 1.90 ± 0.49, which is far greater than 0.4. Hence, the predominance of hydrocarbons from terrestrial origin is confirmed.

#### 3.5.5. Long Chain Hydrocarbons/Short Chain Hydrocarbons (LHC/SHC)

The *n*-alkanes ≤ nC_26_ are referred to as short chain hydrocarbons (SHCs) whereas those above nC_26_ are regarded as the long chain hydrocarbons (LHCs). While SHCs are usually from benthic algal and plankton origin, the LHCs are from vascular plants. The LHC/SHC ratio is usually calculated to assess the abundance of phytoplankton and/or vascular plants in the coastal environments [105].


LHC = C_27_ + C_29_ + C_31_ (2)

SHC = C_15_ + C_17_ + C_19_ (3)

Dominance of phytoplankton is established when the ratio falls within the range of 0.21–0.80; values between 2.38 and 4.33 indicate a mixed origin. The dominance of terrestrial plant waxes is also identified with ratio >4 [106]. In the study, the LHC/SHC ratio for sediments ranged between 0 and 5.11, with an average of 2.15 ± 0.41, an indication of mixed sources.

#### 3.5.6. Average Carbon Length (ACL)

This index is another essential tool used to evaluate the abundance of odd carbon per molecule in the sediment samples collected with a view to establish connection with higher plants n-alkanes. It reveals the average chain length of hydrocarbons presumed to be more persistent in an environment and the presence of heavier hydrocarbons is usually indicated by higher values of ACL. The index is calculated with the expression below [52,107]:

(4) $$\mathrm{ACL}\;\mathrm{value}=\frac{25\left({\mathrm{nC}}_{25}\right)+27\left({\mathrm{nC}}_{27}\right)+29\left({\mathrm{nC}}_{29}\right)+31\left({\mathrm{nC}}_{31}\right)+33\left({\mathrm{nC}}_{33}\right)}{\left(C_{25}+C_{27}+C_{29}+C_{31}+C_{33}\right)}$$

The values fluctuate with lower figure in the area where petroleum hydrocarbon contamination is apparent (Table 9). However, it is always constant in the non-polluted region [108,109]. ACL values in the Algoa Bay sediments varied between 25.97 and 29.44 with an average of 28.07 ± 0.19. The mean values in all the sampling stations revolved around the general average of 28.07 with a very little variation. This implies that the petroleum hydrocarbon contamination in this area is minimal. Statistical analysis of results did not present any significant correlation between CPI and ACL. Therefore, it could be affirmed that increase in CPI does not bring about a corresponding increase in ACL [52,108]. With mean CPI of 1.59, a mixture of petrogenic and marine microorganisms and/or recycled organic matter inputs in the bay sediment samples is suggested [108].

## 4. Conclusions

The present investigation evaluated various ratios and indices to assess the level and sources of petroleum hydrocarbon contamination in Algoa Bay water and sediment samples. Physicochemical parameters were generally within the acceptable levels and the UCM that indicates the presence of hydrocarbons from petrogenic origin was quite low. CPI values in the neighbourhood of 1, however suggest a mixed contribution of hydrocarbons from both anthropogenic and natural sources. The data gathered suggest that the level of hydrocarbons recorded both in the water and sediment matrices are more significantly from industrial and domestic wastes discharge, stormwaters, urban runoff and other anthropogenic sources other than oil spillage. The pollution level is generally adjudged to be minimal, nevertheless there is need for frequent evaluation and strict enforcement of the environmental laws relating to occasional oil spillage and waste disposal in the study site. 

## Figures and Tables

**Table 1:** Description of the study area.

Study Site	Stations	Latitude	Longitude	Description
Algoa Bay	A1	33.8962° S	25.70228° E	Sheltered Bay: Receives industrial effluents, stormwater and runoffs from Motherwell, Markman Canals, Chatty River and sewage outfall from Uitenhage/Despatch Sewage Treatment Works through the Swartkops estuary, which experiences many urban activities and is described as one of the most threatened freshwater systems in South Africa. The water coming from Swartkops River is unsuitable for human consumption. Sheltered bay also takes delivery of stormwater from Port Elizabeth Harbour [31,40,41].
	A2	33.8262° S	25.75429° E	St Croix: Exists between the mouths of the Swartkops and Sundays Rivers, a few kilometres away from the Coega River Mouth. It is a breeding ground for many seabirds and marine mammals, including the African Penguin, Cape Gannet, Cape Cormorant, Roseate Tern, Whitebreasted Cormorant, Kelp Gull, Swift Tern, and Damara Tern, most of which are regarded as threatened and endangered species. Ships are required to pass between St. Croix and the Rij Bank [31,33,40]. Both St Croix and Bird islands are the principal colonies for the African penguin in southern Africa and the established place where Roseate terns reproduce in South Africa [39].
	A3	33.7681° S	25.90603° E	Sundays Estuary: Receives influx from Sundays River, which largely supports many farms with its water [39,42,43].
	A4	33.7321° S	26.06397° E	Alexandria Dune Fields: It is among the largest vegetated and mobile dune fields that exist across the globe. It is located in the north east of the Sundays River mouth, very close to Colchester village [36].
	A5	33.7567° S	26.23478° E	Woody Cape: A section of the Addo National Park, which receives accumulation of marine debris along the coast from Port Elizabeth and the recently built Coega harbour and other industrial activities in the area. Woody Cape is very close to Bird Island and it witnesses trawl fishing occasionally [44].

**Table 2:** Integration events and calibration settings for TPH.

**Integration Events**		
**Time**	**Integration Events**	**Value**
Initial	Slope sensitivity	100
Initial	Peak width	0.04
Initial	Area reject	1
Initial	Height reject	1
Initial	Shoulders	Off
0.242	Integration	Off
3.780	Baseline hold	On
3.780	Area sum slice	Start
3.780	Integration	On
41.500	Integration	Off
41.500	Area sum slice	End
**Calibration Settings**		
**Default RT windows**		
Reference peaks	0.00 min + 5.00%	
Other Peaks	0.00 min + 1.50%	
**Default calibration curve**		
Type	Linear	
Origin	Include	
Weight	Equal	
Calculate uncalibrated peaks	Using n-C_15_	

Min: minutes.

**Table 3:** Seasonal and overall average values for the physicochemical parameters in the water samples.

Parameters	Water Level	Summer	Autumn		Winter	Range	General Average	Guidelines	References
		February	March	May	June				
pH	Surface	8.4 ± 0.002	8.5 ± 0.002	8.8 ± 0.002	8.8 ± 0.08	8.3–8.9	8.6 ± 0.02	5.0–8.0	[64]
	Bottom	8.2 ± 0.001	8.5 ± 0.002	8.7 ± 0.002	8.8 ± 0.001	8.2–8.8	8.5 ± 0.001	5.0–9.0	[63]
Temperature (°C)	Surface	21 ± 0.02	20 ± 0.003	17 ± 0.01	17 ± 0.01	17–22	19 ± 0.01	15–35	[63,64]
	Bottom	16 ± 0.01	19 ± 0.004	16 ± 0.02	17 ± 0	15–19	17 ± 0.01		
Conductivity (μS/m)	Surface	49.11 ± 0.04	48.49 ± 0.01	45.67 ± 0.01	45.57 ± 0.01	45.39–49.9	47.21 ± 0.02	-	
	Bottom	43.7 ± 0.02	47.04 ± 0.01	44.55 ± 0.03	45.23 ± 0	43.07–47.73	45.13 ± 0.01		
Turbidity (NTU)	Surface	1.45 ± 0.84	1.45 ± 0.84	1.33 ± 0.04	1.51 ± 0.03	1.09–1.99	1.43 ± 0.44	0.5–10	[67,68]
	Bottom	2.03 ± 1.17	1.91 ± 1.1	2.09 ± 0.03	1.74 ± 0.04	1.82–3.93	1.94 ± 0.59		
Salinity (PSU)	Surface	35.25 ± 0.03	35.39 ± 0.01	35.36 ± 0.01	35.38 ± 0.01	35.13–35.60	35.34 ± 0.02	-	
	Bottom	35.17 ± 0.02	35.34 ± 0.01	35.31 ± 0.03	35.36 ± 0.001	35.16–35.39	35.3 ± 0.01		
Dissolved Oxygen (mg/L)	Surface	8.38 ± 0.01	6.68 ± 0.03	6.74 ± 0.03	7.32 ± 0.03	5.51–8.96	7.28 ± 0.03	-	
	Bottom	5.2 ± 0.04	6.46 ± 0.01	5.95 ± 0.04	7.42 ± 0.24	4.43–7.76	6.26 ± 0.03		
Chlorophyll (μg/L)	Surface	0.98 ± 0.1	1.29 ± 0.02	0.84 ± 0.06	1.52 ± 0.37	0.55–2.54	1.16 ± 0.14	-	
	Bottom	2.11 ± 0.1	1.68 ± 0.02	1.38 ± 0.03	2.14 ± 0.11	0.64–4.29	1.83 ± 0.06		

**Table 4:** Matrix of Pearson correlation among water quality parameters.

	pH	Temp	Cond	Turb	Sal	DO	Chlor	Depth	TPH
pH	1								
Temp	0.019	1							
Cond	0.028	0.998 **	1						
Turb	0.111	−0.331 *	−0.341 *	1					
Sal	0.255	0.215	0.272	−0.237	1				
DO	0.549 **	0.555 **	0.558 **	−0.222	0.262	1			
Chlor	0.131	−0.245	−0.246	0.105	−0.049	−0.045	1		
Depth	−0.161	−0.648 **	−0.651 **	0.409 **	−0.239	−0.494 **	0.383 **	1	
TPH	0.042	−0.014	−0.019	0.053	−0.074	−0.053	0.013	0.100	1

** Correlation is significant at the 0.01 level (2-tailed). * Correlation is significant at the 0.05 level (2-tailed).

**Table 5:** Mean concentrations of hydrocarbons in the Algoa Bay water (μg/L) and diagnostic ratios.

Parameters	Level	Summer	Autumn		Winter	Range	Overall Average
		February	March	May	June		
**∑(*n*-alkanes)**	Surface	186.53	118.35	77.39	76.47	45.07–273	118.11 ± 13.83
	Bottom	146.46	112.53	125.40	88.15	55.72–307	118.14 ± 13.66
**UCM**	Surface	ND	ND	5.88	59.90	2.28–59.90	23.88 ± 7.02
	Bottom	22.63	ND	ND	10.35	10.35–22.63	16.49 ± 1.94
**Total HCs**	Surface	186.53	118.35	79.74	88.45	45.07–273	121.52 ± 14
	Bottom	150.99	112.53	125.40	90.22	55.72–307	119.79 ± 13.57
**∑(C_15_–C_19_)**	Surface	ND	12.02	2.89	3.68	ND–16.91	4.9 ± 1.13
	Bottom	ND	16.14	8.35	3.96	ND–36.13	7.58 ± 1.89
**∑(C_18_–C_22_)**	Surface	42.08	8.81	2.51	2.09	1.13–63.92	13.13 ± 4.13
	Bottom	43.17	10.89	3.74	2.47	ND–72.97	14.36 ± 4.2
**∑(C_25_–C_35_)**	Surface	47.83	1.30	34.9	40.63	ND–169	29.98 ± 8.23
	Bottom	10.89	1.07	75.29	53.55	ND–237	35.47 ± 12.23
**L/H**	Surface	1.14	8.53	0.11	0.16	0.06–13.28	2.49 ± 0.92
	Bottom	1.76	7.23	0.17	0.13	0.07–14.09	2.32 ± 0.80
**U/R**	Surface	0	0	0.03	0.11	0–0.53	0.04 ± 0.03
	Bottom	0.04	0	0	0.04	0–0.20	0.02 ± 0.01

UCM: unresolved complex mixture; HCs: hydrocarbons; L/H: low molecular n-alkanes/high molecular *n*-alkanes; U/R: unresolved/resolved; ND: not detected.

**Table 6:** Average concentration of hydrocarbons in the bay sediment (mg/kg) and sources diagnostic ratios.

Parameters	Summer	Autumn		Winter	Range	Overall Average
	February	March	May	June		
∑(*n*-alkanes)	4.23	14.55	14.95	17.17	0.72–27.03	12.72 ± 1.74
UCM	ND	ND	0.23	ND	0.17–0.27	0.23 ± 0.01
Total HCs	4.23	14.55	15.13	17.17	0.72–27.03	12.77 ± 1.74
∑(C_15_–C_19_)	0.08	1.8	0.47	0.70	ND–2.22	0.76 ± 0.17
∑(C_18_–C_22_)	1.00	1.03	1.24	1.25	ND–1.91	1.13 ± 0.09
∑(C_25_–C_35_)	0.84	1.79	3.23	7.29	0.39–11.83	3.29 ± 0.76
LHC/SHC	2.39	0.49	2.57	3.53	0–5.11	2.15 ± 0.41
L/H	1.05	0.63	1.14	0.24	0–2.92	0.77 ± 0.15
C_31_/C_19_	0.76	0.23	1.82	0	0–7.41	1.90 ± 0.49
U/R	0	0	0.01	0	0–0.020	0.003 ± 0.002
CPI	1.66	1.18	1.28	1.62	0–2.15	1.59 ± 0.11
ACL	27.78	27.77	28.15	28.58	25.97–29.44	28.07 ± 0.19

UCM: unresolved complex mixture; HCs: hydrocarbons; LHC/SHC: long chain hydrocarbons/short chain hydrocarbons; L/H: low molecular *n*-alkanes/high molecular *n*-alkanes; U/R: unresolved/resolved; CPI: carbon preference index; ACL: average carbon chain length; ND: not detected.

**Table 7:** Percentage moisture, organic carbon and organic matter contents of the sediments.

	% Moisture	% Organic Carbon	% Organic Matter
Range	13–27.96	1.06–2.05	1.82–3.53
Mean	22.96 ± 3.25	1.49 ± 0.30	2.56 ± 0.51

**Table 8:** Pearson correlations among the sediments quality parameters.

	% Moisture	% OC	% OM	TPH
% Moisture	1			
% OC	0.595 *	1		
% OM	0.628 *	0.446	1	
TPH	0.028	0.270	−0.196	1

* Correlation is significant at the 0.05 level (2-tailed). % OC: percentage organic carbon; % OM: percentage organic matter.

**Table 9:** Molecular ratios and indexes for source identification of hydrocarbons in the coastal water and sediment.

Ratios/Indexes	Biogenic Origin (Plants/Microorganisms)			Petrochemical/Anthropogenic Origin	
	Terrestrial	Mixed	Marine	Degraded Oil	Fresh Oil
CPI	>1 (mostly 3–10)	-	~1	~1 or <1	-
U/R	-	-	-	>4	-
ΣLMW/ΣHMW	<1	-	~1	~1	>2
nC_31_/nC_19_	>0.4	-	≤0.4	-	-
LHC/SHC	>4	2.38–4.33	0.21–0.80	-	-
ACL	Higher & Constant (close range)			Depletes & Fluctuates (wide range)	

CPI: carbon preference index; U/R: unresolved/resolved; ΣLMW/ΣHMW: low molecular weight *n*-alkanes/high molecular weights alkanes; LHC/SHC: long chain hydrocarbons/short chain hydrocarbons; ACL: average carbon chain length.
